# A Roadmap to Newborn Screening for Duchenne Muscular Dystrophy

**DOI:** 10.3390/ijns3020008

**Published:** 2017-04-07

**Authors:** Samiah A. Al-Zaidy, Michele Lloyd-Puryear, Annie Kennedy, Veronica Lopez, Jerry R. Mendell

**Affiliations:** 1 Department of Pediatrics, Divisions of Neurology and Neuromuscular at Nationwide Children’s Hospital, Columbus, 43205 OH, USA;; 2 Parent Project Muscular Dystrophy, Hackensack, 07601 NJ, USA;; 3 Mark Krueger & Associates, Inc., New York, 10175 NY, USA;; 4 Center for Gene Therapy, Research Institute, Nationwide Children’s Hospital, Columbus, 43205 OH, USA

**Keywords:** Duchenne, newborn screening, gene therapy, muscular dystrophy

## Abstract

Duchenne muscular dystrophy (DMD) is the most common childhood form of muscular dystrophy, with an estimated frequency of 1:5000 live births. The impact of the disease presents as early as infancy with significant developmental delays, and ultimately loss of ambulation and respiratory insufficiency. Glucocorticoids are the only pharmacological agents known to alter the natural progression of the disease by prolonging ambulation, reducing scoliosis, and assisted ventilation. Introduction of therapy at an early age may halt the muscle pathology in DMD. In anticipation of the potentially disease-modifying products that are reaching regulatory review, Parent Project Muscular Dystrophy (PPMD) formally initiated a national Duchenne Newborn Screening (DNBS) effort in December 2014 to build public health infrastructure for newborn screening (NBS) for Duchenne in the United States. The effort includes a formalized national Duchenne Newborn Screening Steering Committee, six related Working Groups, a Duchenne Screening Test Development Project led by PerkinElmer, a program with the American College of Medical Genetic and Genomics’ Newborn Screening Translation Research Network (NBSTRN), and collaborations with other Duchenne partners and federal agencies involved in NBS. We herein review the organization and effort of the U.S. DNBS program to develop the evidence supporting the implementation of NBS for DMD.

## 1. Statement of Problem

### Background

Duchenne muscular dystrophy (DMD) is the most prevalent pediatric form of muscular dystrophy, with an incidence of 1:5000 male births [1]. The disabling progression of the disease is a result of the absence of the dystrophin protein caused by mutations in the *DMD* gene [2]. The disease is inherited as an X-linked recessive disorder with an estimated rate of 33% de novo mutations in both maternal carriers as well as affected males [3,4]. While a disabling mutation of dystrophin causes DMD in males, it can manifest across a range of severity in heterozygous females; hypomorphic *DMD* mutations cause Becker muscular dystrophy—milder form of dystrophinopathy, but otherwise similar in many respects [5]. Therefore, there may be no prior history of the disease in the family.

The cardinal features of skeletal muscle weakness generally do not manifest until school age, and hence diagnosis is not pursued in newborns unless a family history suggests enhanced risk for the disorder. About half of affected boys manifest a delay of motor milestones [6]. The disorder is associated with impaired learning to a variable degree, and is associated with progressive cardiomyopathy not tightly associated with skeletal muscle involvement. Downstream complications of DMD restrict independent mobility, are associated with scoliosis and other skeletal deformity, and inevitably lead to progressive respiratory insufficiency.

The progressive nature of the disease presents a significant emotional and psychological burden, and despite advances in the knowledge of the disease and emerging therapies, the diagnostic odyssey remains a challenge for most families. DuchenneConnect, the online self-report patient registry established by Parent Project Muscular Dystrophy (PPMD), showed that the mean age of diagnosis is approximately 4 years (±2.3) [7], corroborating findings from earlier surveillance studies by the Center for Disease Control and Prevention’s (CDC) Duchenne/Becker Muscular Dystrophy (DBMD) surveillance program, MD STARnet [8]. However, onset of symptoms precedes the diagnosis by years, and is clinically under-recognized. MD STARnet identified a 2–5 year average diagnostic delay from the time parental concerns were first reportedly voiced to a primary care physician to the date of confirmed DMD diagnosis [9]. Compelling data of delayed neurodevelopmental milestones including motor, cognitive, and speech were described in DMD boys as early as infancy, supporting the earlier onset of disease manifestations [10–12]. These observations were validated by significant delays observed on the Bayley III scale in DMD infants compared to healthy controls [13].

Since its inception, PPMD and its constituency have focused on the need to identify children with DMD at an early age. PPMD is the world’s largest patient-advocacy organization focused on ending Duchenne muscular dystrophy. PPMD and their partners have worked tirelessly to build a robust therapeutic pipeline, regulatory infrastructure, and clinical care network through collaborations with U.S. Congressional leaders, federal agencies, clinical and research experts, and the pharmaceutical industry. In preparation for establishing a system of early identification of infants with DMD, PPMD set out to address the research needed for effective treatments, including funding basic research as well as clinical trials and engaging the U.S. Food and Drug Administration (FDA) throughout their approval processes, and educating families and health care professionals. In 2009, CDC and PPMD launched an effort to address the delay that families frequently experience between symptom onset and diagnosis of neuromuscular disorders through the formation of the National Task Force for Early Identification of Childhood Neuromuscular Disorders. While this effort yielded a robust clinical resource (www.childmuscleweakness.org) and awareness campaign, it did not significantly impact the diagnostic delay within DMD [14]. The latter activities also included the development of treatment guidelines and education of health care professionals in the care and treatment of DMD. However, because delay in diagnosis was not significantly impacted, PPMD moved to focus on the early identification of newborns with DMD through state-based newborn screening (NBS) programs. Perhaps even more compelling was the progression of the Duchenne therapy development pipeline, resulting in dozens of late-stage human clinical trials nearing regulatory review, which would further strengthen the rationale for the need of an NBS program. Currently, corticosteroid therapy slows the progression of skeletal muscle weakness and the associated functional complications [15], but the past decade has witnessed progress in the development of a range of different approaches to therapy. As all current therapeutic strategies aim at slowing progression rather than restoring lost functions, the introduction of population-based NBS for DMD offers the best approach for early identification of those who could most benefit from these therapies.

Because PPMD has fostered strong collaborations with both private and public partners around DMD clinical infrastructure and resource development, the advocacy organization was well-positioned to serve as a convener of an effort focused on developing a public health system for DMD NBS. This effort brought together all U.S. federal agencies committed to NBS along with other relevant stakeholders.

In the U.S., programs for the screening of newborns reside within state-based NBS programs [16]. These programs provide a universal and systematic approach to NBS in each state, and have been in existence in most states for over 50 years [17,18]. Six components characterize NBS programs: screening, diagnosis, treatment and management, follow-up, education, and quality assurance [16]. State programs considering NBS for a condition must have or be able to build the infrastructure for each component for that condition.

## 2. Achieving the Goal of State-Based Universal NBS

Although there are federal guidelines for NBS, each state program is controlled by its own policies and procedures. Currently, the Secretary’s Advisory Committee on Heritable Disorders and Genetic Diseases in Newborns and Children (ACHDNC) established by the Department of Health and Human Services (DHHS) in 2003 advises and guides the Secretary of DHHS regarding the “most appropriate application of universal NBS tests, technologies, policies, guidelines, and programs in order to effectively reduce morbidity and mortality in newborns and children who have or who are at risk for heritable disorders” [19]. Advice and guidance—if accepted by the Secretary—may become accepted federal policy, or if concerning screened conditions, may become part of the Secretary’s recommended uniform screening panel (RUSP). State public health NBS programs have generally accepted the recommendations from the Secretary. The path to nomination and placement on the RUSP encompasses multistep processes: submission, evaluation of readiness for ACHDNC review, evidence review, and ACHDNC decision submission to the Secretary of DHHS, if accepted. Pilot studies usually precede the formal implementation of changes to the NBS panels.

**Figure 1.**
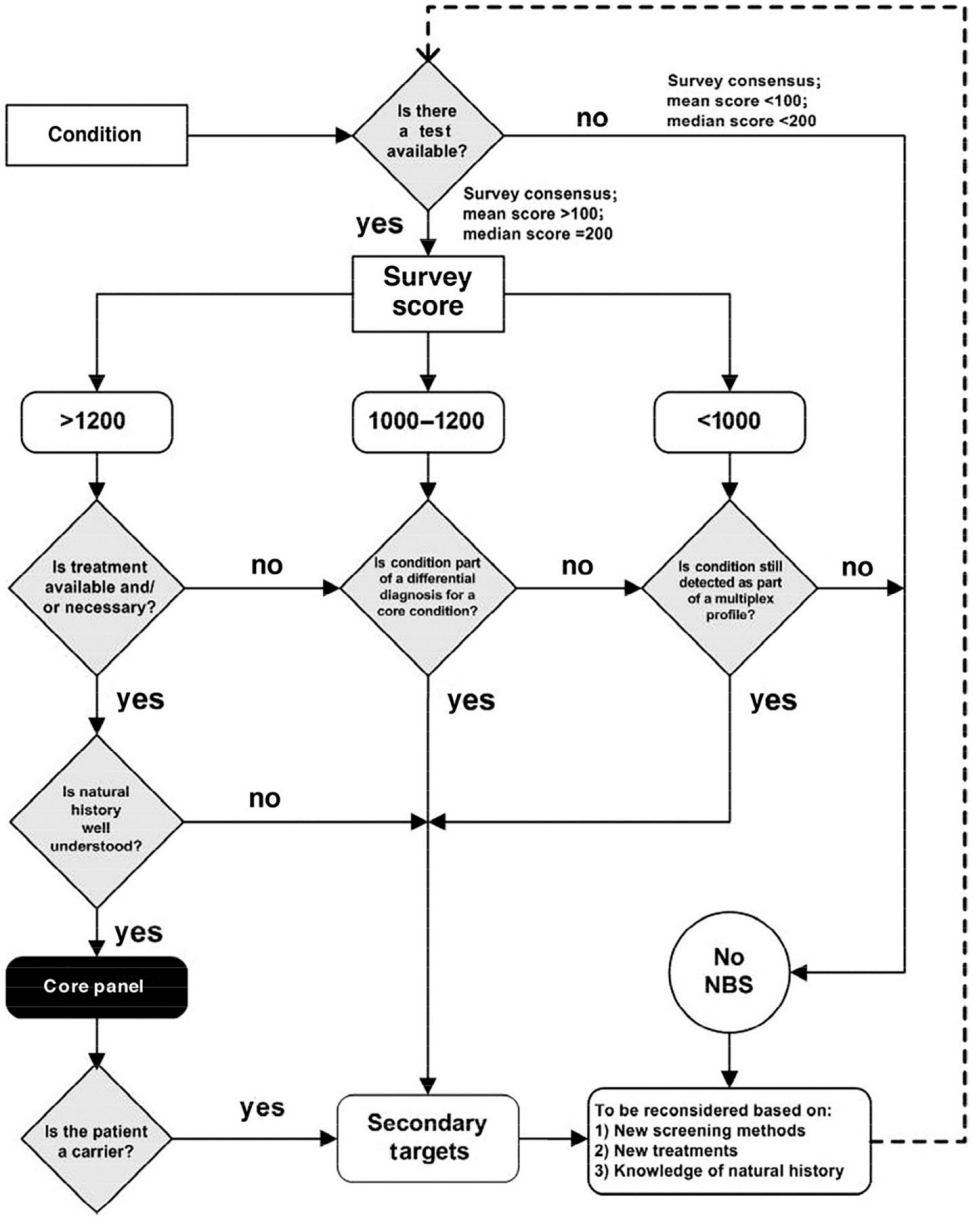
Newborn screening (NBS) algorithm. Reproduced with permission from [18]. Copyright 2006 by the AAP.

Considerations in the review process include whether (1) the condition poses a public health problem that justifies screening the whole population, (2) there is a test available with acceptable sensitivity and specificity, (3) the condition can be identified through screening within the first 24–48 h whereas it would not ordinarily be detected, and (4) there are clear benefits to the infant from early detection, be it through available treatment or other timely interventions [16,18]. The latter three specifications were suggested as the minimum criteria required to be added to a NBS battery by the American College of Medical Genetics and Genomics (ACMG) NBS Expert Group in the 2006 report commissioned by the Maternal and Child Health Bureau (Figure 1) [18].

Barriers to acceptance in the review processes may occur at any step along the way, but are generally due to the lack of sufficient evidence for decision-making. Most of the disorders considered for NBS are very rare; the evidence for conditions is often based on case reports and observational studies, knowledge of the “normal” population is limited, and ascertainment is generally based on small sample sizes and is therefore often biased, leading to limited understanding of penetrance, clinical course, and effective treatments.

If the condition under review is accepted to the RUSP, there are also challenges to the successful implementation of screening for the condition in NBS programs. A screening program will be most effective when the appropriate infrastructure exists to support education, sample collection, and laboratory testing, treatment, short-term and long-term follow-up, and system evaluation [16]. Training and education are major challenges related to NBS for Duchenne. Not only is there a lack of training and expertise among health care providers about Duchenne detected through NBS, but health care providers often do not have the necessary tools to educate and guide parents through the decision-making process [16–18]. Budget constraints and laboratory capacity are other challenges directly related to the quality and robustness of NBS programs [16]. With insufficient funding, programs are not properly evaluated and cannot assure comprehensive, sustainable care, including screening for new conditions, inclusion of new screening technologies, and resources for follow-up and treatment [16–18].

Communication among stakeholders involved in NBS also poses a barrier to successful implementation of such programs [17]. In most cases, decisions about the NBS panel are delegated to state health officials, a state board of health, or a genetics or NBS advisory committee. However, state policymakers can lack complete understanding of the criteria to be applied to conditions for consideration or of the testing technology [16]. This lack of awareness coupled with political incentives results in different states—all of which have a statute or regulation that allows or mandates universal NBS—screening for different conditions, inconsistent with the prevalence of the conditions or the scientific evidence supporting testing at birth [16]. Therefore, successful program implementation not only requires state leadership to establish education and training programs for both public health and health care professionals concerning the screening, diagnostic, treatment, and follow-up protocols required, but also the development of quality assurance and evaluation policies to guarantee the appropriate representation of conditions in the state’s NBS battery.

## 3. The Ohio Newborn Screening Pilot

The 2007–2011 NBS pilot study in Ohio provides an excellent model for effectively implementing DMD NBS at a national level. Supported by the CDC, with co-funding from PPMD and the National Institutes of Health (NIH), the Ohio pilot was a four-phase study that validated a two-tier system for conducting DMD NBS: an initial screening for creatine kinase (CK) level (previously validated in 1979 as a biomarker for Duchenne at birth), followed by DNA isolation and *DMD* gene testing on the same dried blood spot [1]. All DNA samples were analyzed for single or multi-exon deletions/duplications in the gene. The *DMD* gene analysis reduces the number of false-positives from the initial screening based exclusively on CK levels, and suggests a path for follow-up with families of newborns with elevated CK results.

Phase 1 of the Ohio pilot established a threshold that would trigger the second-tier DNA analysis by sampling 30,547 consecutive anonymous dried blood spot samples from male and female newborns. Based on the population-based range of CK levels at birth, a threshold of >600 U/L was chosen at three standard deviations above the mean [1]. Phase 2—screening of 6928 newborn males at major birthing hospitals in Columbus and Cincinnati, Ohio—provided the impetus to shift the CK threshold to a higher level of 750 U/L [1]. This new threshold was then validated by the results of the 10,937 newborn males screened state-wide in Phase 3. By increasing the CK threshold from 600 U/L to 750 U/L, the number of newborn males requiring DNA testing was reduced by 68%, and the false positive rate was reduced from 1.6% (108 of 6926) in phase 2 to 0.52% (57 of 10,936) in Phase 3 [1].

During Phase 4, de-identified blood spots from the NBS cards of 19,884 males and 18,763 females were anonymously screened through the Ohio Department of Health (DOH). The goal of this final phase was to further validate the two-tier system by increasing the sample size and to include both genders. Females were included to enhance the chance of identifying mutations in autosomal genes, allowing this two-tier method of screening to account for subjects with elevated CK levels but who were negative for the *DMD* gene mutation. Out of the 308 males and 242 females with CK levels >750 U/L, there were 10 males and 2 females with CK levels ≥2000 U/L; seven of the males and two of the females had no mutations for DMD. For these individuals, mutation analysis was extended to include the seven most common limb-girdle muscular dystrophy (LGMD) genes (*DYSF*, *CAPN3*, *SGCA*, *SGCB*, *SGCC*, *SGCD*, and *FKRP*) [20]. Mutations were found in one female (*DYSF* point mutation) and two males (point mutations in *SGCB* and *FKRP*, respectively). The finding that other muscular dystrophy-related gene mutations can be identified as part of the screening process is important for NBS panels and future studies. It is presumed that other less common muscular dystrophy-related genes could also be identified with more exhaustive testing. Among the 37,649 newborn infants screened during phases 2, 3, and 4 of the pilot, six subjects were identified to have mutations in the *DMD* gene. A significant finding from this study is that all individuals with mutations for DMD had CK values ≥2000 U/L.

The Ohio study validated a cost-effective model with minimal false-positives for the implementation of NBS for Duchenne in the United States. The two-tier system of analysis allows all testing to be completed shortly after birth from the same dried blood spot [1]. It was also noted that there was no loss of enzyme activity in samples analyzed within the first five days after collection, corroborating reports of stability of this enzyme assay at room temperature for up to a week [21]. The screening test adequately fits within current U.S. NBS practices, as it is minimally burdensome and can be completed prior to the discharge of mother and child from the hospital, requiring minimal tracking of newborns for follow-up and diagnostic testing. In addition, the Ohio pilot offered the opportunity to develop a system of follow-up and treatment for those infants identified as screen positive for muscular dystrophies.

## 4. Addressing the Barriers and Building the Infrastructure

In January of 2015, PPMD convened a meeting that included federal partners concerned with NBS and experts in both Duchenne muscular dystrophy and newborn screening to assess the feasibility of NBS for DMD. The 2015 meeting resulted in the establishment of a National Duchenne Newborn Screening Steering Committee and the formation of six working groups to address key issues in the consideration of this condition as a candidate for the Secretary of DHHS’s RUSP. The 2015 meeting examined the way forward and the evidence needed to present DMD NBS for consideration, including the alignment of resources for NBS pilot studies in additional states. The key issues addressed by each working group are broken down in the following.

### 4.1. Outreach and Education of Healthcare Providers and the Patient Community

This working group is comprised of representatives from a variety of leading patient advocacy organizations, health care providers, genetic counselors, federal agency representatives specializing in public health education, and a parent whose child was identified as having DMD through the Ohio DMD NBS pilot. Together, this group is developing educational materials about Duchenne’s and the DMD NBS program for providers ranging from those in state health departments to birthing center personnel and primary care providers. This workgroup is also reviewing existing educational materials for newly-diagnosed DMD families and those with similar conditions, and is recommending modifications and updates so that materials are appropriate for families of newborns.

### 4.2. Laboratory Test Validation and Refinement, including Screening Algorithm Development

This workgroup was asked to evaluate the analytic markers associated with DMD that can be used in population-based screening (specifically CK levels), and to identify and determine the analytical validity of the screening tests that can be used to find these markers. This task includes determining the clinical validity of the available NBS screening test algorithms in dried-blood spots, the evaluation of expected positive predictive value (PPV), and the likelihood that if the test is positive that the infant has DMD. Data extrapolated from the experiences of ten DMD newborn screening programs worldwide in which more than 1.8 million newborns were screened between the years 1975 and 2011 with 344 identified DMD subjects was invaluable for this task [22,23]. All ten programs utilized an enzyme assay based on the total serum CK levels, with variability in the chosen cutoff for CK levels but a shared finding of both false positive tests as well as missed subjects with DMD due to false negative results. False positive cases in these pilot studies included mostly non-DMD muscular dystrophies, as CK elevation is a manifestation of the dystrophic pathology and not specific for the DMD phenotype. Conversely, a false negative finding may miss the X-linked dilated cardiomyopathy—a form of dystrophinopathy. Based on the experience of the DMD NBS program in Wales [24] and individual laboratory experience using enzymatic assays, the DMD Laboratory workgroup concluded that an immunoassay for detecting an elevated CK should be piloted along with the enzymatic assay to determine which approach has appropriate analytical/clinical validity and utility for use by a public health laboratory. An immunoassay recently developed to provide high-throughput detection of the isoform CK-MM showed a high positive association with total CK enzyme activity results obtained from dried blood spots of 10 DMD cases [25]. CK-MM was chosen for this assay due to its higher specificity as a marker for skeletal muscle injury than the total CK enzyme. This assay shares the limitations of other immunoassays; however, it may prove to be superior to enzyme assays, with greater stability at room temperature and a higher specificity that would potentially overcome the high rates of false positives. In an attempt to further refine the first-tier screen for CK to reduce the false positive rate seen in previous Duchenne newborn screening pilots [22], an initiative with the California Department of Health was developed in collaboration with PerkinElmer Incorporated. This project aims at retrieving residual bloodspot specimens obtained from DMD patients from the California Biobank for advancing the development of the screening assay. PPMD is working directly with four major DMD care centers based in California that have agreed to participate in the project and to assist with local institutional research board (IRB) processes and patient informed consent from eligible families. PerkinElmer, Inc. provided PPMD with a grant to support this effort.

### 4.3. Clinical Care Considerations for Pre-Symptomatically Identified Infants with DMD

International care and treatment of patients with Duchenne muscular dystrophy is guided through established care standards published in 2010, and are commonly known as the CDC’s DBMD Care Considerations. The Care Considerations include recommendations for diagnosis and treatment, initiated by clinician or parental concerns or family history.

This working group includes providers, federal agency representatives, and advocacy organization representatives who have been closely involved in the care and management of families identified through previous Duchenne NBS pilots or other infant programs in DMD. Many of the pediatric neuromuscular experts within this workgroup have also been involved in developing and validating outcome measures for infants and toddlers with DMD for use in ongoing therapeutic experimental trials. The group also works to examine approaches to DMD diagnosis available in newborns versus older children and the impact associated with DMD in the quality of life of patients and in family and caregivers when identified through usual care versus newborn screening. Lastly, this working group identifies other specific factors that may affect treatment plan or outcome and the cost of diagnosis through usual care. In addition, this group will evaluate the number of newborns that would be affected by NBS for DMD and which may require short- or long-term follow up services, including true and false positive cases and true and false negative cases. The group will identify the resources required to ensure readiness and feasibility of the state’s NBS programs to adopt screening and follow-up services for DMD and evaluate the availability and accessibility of the resources required to ensure the capacity of the health service system to implement screening and short- and long-term follow-up resulting from expanded newborn screening (diagnosis, treatment, follow up) [22].

### 4.4. Long-Term Follow-Up

This working group is tasked with identifying intermediate or proximal outcome measures and biomarkers that can be used to monitor and evaluate the status of DMD, as well as examining the association between these intermediate outcomes and health outcomes. This group seeks to answer whether interventions for DMD detected through NBS lead to an improvement in intermediate measures compared to clinical detection, and whether factors other than age of initiation modify the effect of treatment on intermediate measures.

### 4.5. Bioethical, Social, and Legal Considerations

This working group examines the benefits to the child and the family associated with pre-symptomatic identification of DMD, independent of the timing of treatment. Included in this analysis is the extent by which the observed incidence or spectrum of DMD changes compared to clinical detection, and whether screening for DMD can detect other conditions. This group considers the physical and psychosocial harms associated with screening outcomes, such as false-negatives, false-positives, and DMD carrier status, as well as the strategies that can minimize these harms. In particular, this group has identified ethical, legal, and social issues/concerns that need to be addressed in any pilot examining newborn screening for a particular condition.

### 4.6. Evidence Review

Using the condition nomination form for ACHDNC review and the ACHDNC evidence review process as a guideline, this working group is examining the cumulative evidence put forth by the other five working groups, as well as the evidence existing within publications related to the DMD space. Once evidence gaps are identified, working group members work pro-actively with DMD NBS program leadership to generate data with methodological rigor to support the nomination of Duchenne to the RUSP.

## 5. Path Forward: Assessment of Current Treatments and Next Steps

As indicated previously, necessary components for screening a condition at birth include a screening test with sound clinical and analytical utility and validity to detect that condition and a safe and effective treatment for that condition.

### 5.1. Current Treatments

The only current treatment known to alter the natural history of DMD is administration of glucocorticoids. Evidence for glucocorticoid-induced improvement was reported nearly two decades ago through a double blind, randomized controlled trial in a large cohort (>100 subjects) of DMD subjects. Efficacy was established using two doses of prednisone (0.75 mg/kg vs. 1.5 mg/kg) [26], and muscle strength and functional outcomes were significantly improved compared to placebo (*p* < 0.001). Similar results were later reported with deflazacort (0.9 mg/kg/day)—an alternative sodium-sparing corticosteroid [27]. A weekend dosing regimen (10 mg/kg/wk) showed equal efficacy to daily steroids with advantages because of fewer side effects and preserved bone lengthening [28]. Additional follow-up studies reported that corticosteroids prevent scoliosis and extend independent ambulation compared to untreated DMD patients [29].

### 5.2. Disease-Modifying Approaches Nearing Approval

Exon skipping is an approach to gene repair that targets the pre-mRNA transcript, introducing alternative splice sites that result in skipping one or more targeted exons and subsequent restoration of the dystrophin reading frame. This strategy is estimated to potentially benefit 90% of DMD patients [30]. In a Phase I proof of principle study, muscle samples from subjects treated with a morpholino oligomer (AVI-4658/eteplirsen) skipping exon 51 of the DMD gene showed restoration of dystrophin expression [31]. Subsequent Phase I/II studies extending over 4 years confirmed functional improvement in treated subjects demonstrated by prolonged ambulation and a reduced rate of decline in the six-minute walk test (6MWT) compared with natural history controls [32]. This is the only drug to ever show increased dystrophin expression and functional improvement, and the first treatment to have received accelerated approval by the FDA for DMD.

In a similar approach to exon skipping, stop codon readthrough is also based on gene mutation, and could be applied to up to 13% of DMD subjects to produce a full-length protein from pathogenic premature stop codons. Ataluren is a small molecule developed by PTC Therapeutic to advance an orally bioavailable drug that selectively reads through disease-causing nonsense mutations with no off-target effects on normal stop codons. Preclinical efficacy was demonstrated by the restoration of dystrophin production in skeletal and cardiac muscles of treated *mdx* mice within 2–8 weeks of treatment [33]. This enabled a Phase III multicenter randomized double-blind placebo-controlled study to determine the efficacy and safety of Ataluren in DMD boys 7–16 years of age with known stop codons. The study is currently underway, but initial results announced in late October 2015 showed a strong safety profile with a modest improvement of 15 meter above baseline 6MWT in the study population (*n* = 228). Ataluren is currently available in some European countries, but was not granted approval by the FDA.

In an attempt to circumvent the limitations of gene editing, an alternative strategy would be gene replacement. The major hurdle to this approach in DMD subjects is the large size of the *DMD* gene that exceeds the packaging capacity of the widely used adeno-associated (AAV) viral vector. To overcome this, miniature versions of the *DMD* gene are in development that would allow packaging into the rAAV and amelioration of the clinical phenotype. Pre-clinical studies in the *mdx* mouse using a mini-dystrophin gene under the control of a cytomegalovirus (CMV) showed robust and sustained dystrophin expression and increased resistance of treated muscle fibers to contraction-induced injury [34,35]. These studies set the stage for translation to clinic; a Phase I safety study delivering a micro-dystrophin gene is currently in progress at Nationwide Children’s Hospital.

### 5.3. Future Landscape (CRISPR)

The most recent breakthrough in genome engineering is the CRISPR/Cas9 system that allows gene editing with promise for clinical translation. The clustered regularly interspaced short palindromic repeats system—specifically CRISPR/Cas9—can be delivered by AAV, serving as a novel vector for gene therapy in DMD [36–38]. Researchers have utilized the CRISPR/Cas9 system to target a point mutation in exon 23 of the *mdx* mouse model that results in a premature stop codon. With this technique, both histological and functional improvement occurred; a reduction in infiltrating inflammatory cells and skeletal muscle fibrosis was observed, as well as improved force generation and increased grip strength in treated mice. An important highlight in the outcome of these studies is the recovery of dystrophin expression in cardiac muscle cells. Collectively, these *mdx* mouse results provided proof of principle for a promising cure if implemented in early stages of the disease at a young age.

## 6. Conclusions

It is clear that a multi-front line of attack—as is currently being applied for DMD—will change the landscape for NBS. The two-tier system has wide applicability, and is currently used in the screening for other disorders (sickle cell diseases and cystic fibrosis). Plans for moving forward using this approach fit within current U.S. NBS practices because it is minimally burdensome and can be completed prior to the discharge of mother and child from the hospital. Refinements in the program are underway, focusing on the first-tier screen for CK levels to reduce the false positive rate seen in previous DMD newborn screening pilots. Efforts are also underway to ensure readiness and feasibility of NBS programs to adopt follow-up services for DMD that address the applicability of the treatments that are on the horizon. Bioethical, social, and legal implications are currently being closely examined to address the impact of pre-symptomatic identification of DMD, independent of the timing of treatment. Improvement in disease-modifying approaches like glucocorticoids, exon skipping, and agents reducing fibrosis, to the more aggressive strategies like gene replacement and CRISPR/Cas9 provide promise that the dystrophic condition can be circumvented shortly after birth before significant muscle fiber loss and severely scarred muscle limit the benefit of therapy. The ongoing efforts for treatment of Duchenne have dramatically accelerated the efforts for moving ahead with NBS. The existing diagnostic model for DMD with children being diagnosed on average by age 4—after a 3- to 5-year diagnostic odyssey—now denies clinicians the opportunity to offer families potentially life-altering medical interventions in a timely manner. DMD NBS is no longer an anticipatory program; it has now become a public health issue.
